# Complete Genome Sequences of Three Listeria monocytogenes Bacteriophage Propagation Strains

**DOI:** 10.1128/MRA.01159-20

**Published:** 2021-01-07

**Authors:** Lauren K. Hudson, Tracey Lee Peters, Daniel W. Bryan, Yaxiong Song, Henk C. den Bakker, Thomas G. Denes

**Affiliations:** a Department of Food Science, University of Tennessee, Knoxville, Tennessee, USA; b Center for Food Safety, University of Georgia, Griffin, Georgia, USA; Queens College

## Abstract

Bacteriophages can be used as a biocontrol for the foodborne pathogen Listeria monocytogenes. Propagation of phages is a necessary step for their use in experimental studies and biocontrol applications. Here, we present the complete genomes of three Listeria monocytogenes strains commonly used as propagation hosts for *Listeria* phages.

## ANNOUNCEMENT

*Listeria* phages are suitable for use as biocontrols in food safety applications (1–6), can be used for the detection and subtyping of *Listeria* (7–9), and are studied for their role in the ecology and evolution of the foodborne pathogen Listeria monocytogenes (10–13). To work with *Listeria* phages, they must be propagated to sufficiently high titers, preferably using a bacterial propagation host that lacks prophages (14) to limit contamination of the stock with unwanted temperate phages or prophage-derived particles (15). Here, we present the complete genome sequences of three widely used L. monocytogenes phage propagation host strains, Mack (FSL F6-0367) (13, 16–24), FSL J1-0175 (11, 13, 17, 25–27), and FSL J1-0208 (11, 17, 23, 26, 28–36), which represent serotypes 1/2a, 1/2b, and 4a, respectively. These strains were obtained from Martin Weidman (Cornell University). The genomes of FSL J1-0175 and FSL J1-0208 have previously been published (GenBank assembly numbers GCA_000168415.1, GCA_000168435.1, and GCA_000250715.1) (28) but were not complete.

All strains were cultivated in brain heart infusion broth overnight at 37°C with shaking. Genomic DNA was extracted using a Qiagen DNA Easy minikit (Hilden, Germany) per the manufacturer’s instructions. Illumina libraries were prepared using a Nextera XT kit, and sequencing was performed using a NextSeq 550 instrument (150-bp paired-end reads). Nanopore libraries were prepared using the Oxford Nanopore rapid barcoding kit (SQK-RBK0004), and sequencing was performed using a MinION FLO-MIN106 flow cell; Guppy v3.2.10 (fast model) was used for base calling, and EPI2ME v2019.11.11 was used to demultiplex and trim the barcodes. Illumina reads were trimmed using Trimmomatic v0.35 (37) (with the parameters ILLUMINACLIP:NexteraPE-PE.fa:2:30:10 LEADING:3 TRAILING:3 SLIDINGWINDOW:4:15 MINLEN:36). Hybrid genome assemblies were created with Unicycler v0.4.8-beta (38) (with pilon polishing), which automatically resolves overlaps and circularizes and reorients the assembly to begin at the *dnaA* gene. Assemblies were annotated using the NCBI Prokaryotic Genome Annotation Pipeline (PGAP) v4.13 and were queried for acquired antibiotic resistance genes using ResFinder v4.0 (39). Default parameters were used except where otherwise noted.

The sequencing read and genome assembly statistics are presented in Table 1. All genomes were assembled into single-contig complete chromosomes of 2.78 to 2.96 Mb; FSL J1-0208 had an additional contig representing a plasmid, which was previously described (28). The Mack genome was very similar (≥99.97% ANIm [average nucleotide identity calculated using MUMmer] over ≥99.96% of the genome as calculated with JSpeciesWS [40], and ≤16 single nucleotide polymorphism [SNP] differences as inferred by kSNP3 [41]) to four other published genomes (NCTC7973 [GenBank assembly number GCF_900637785.1] [42], WSLC1001 [GCF_000568475.1] [43], EGD [GCF_000582845.1] [44], and SLCC5850 [GCF_000307045.1]), which strongly suggests that they descended from the same isolate(s) originally isolated by E. G. D. Murray (45). All three genomes contained 1 to 3 prophage regions, which were most similar to parts of temperate phages LP-101 (23), A118 (46), and PSA (47), and lytic phages LP-064 and LP-125 (23, 48) (Table 1). Although the prophage regions detected were designated questionable or incomplete, their presence should be considered, as prophage elements may excise from the genome during phage amplification. The *fosX* gene (92.56 to 97.26% identity), which is related to fosfomycin resistance (49, 50), was detected in all three genomes. Thus, precaution should be taken in commercial use of these strains as phage propagation hosts.

**TABLE 1:** Sequencing and assembly statistics for L. monocytogenes strains

Strain (BioSample no.)	Serotype	MLST ST^*a*^		Sequencing read data^*c*^					Assembly^*f*^ and annotation data										Prophage region data^*h*^			
				Type^*b*^	SRA run no.	No. of reads	Avg. read length (bp)		Genome location^*d*^	GenBank accession no.	Length (bp)	G+C content (%)	Avg Illumina coverage (×)	No. of total genes	No. of total CDS^*e*^	No. of coding genes	No. of RNA genes^*g*^		Position	Length (kb)	Completeness^*i*^	Most common phage hit (GenBank accession no.; no. of hits)
FSL J1-0175 (SAMN16231352)	1/2b	87		I	SRR12695186	3,413,542	136.48		C	CP062129	2,957,228	38.01	153.3	2,951	2,862	2,843	89		1–23761	23.7	Q	LP-101 (NC_024387; 20)
				N	SRR12695182	289,800	5,590.76												2397961–2408689	10.7	Q	A118 (NC_003216; 5)
FSL J1-0208 (SAMN16231353)	4a	569		I	SRR12695185	3,398,910	135.55		C	CP062127	2,781,474	38.00	153.9	2,873	2,784	2,757	89		130809–141536	10.7	I	A118 (NC_003216; 6)
				N	SRR12695181	238,635	5,276.33		P	CP062128	77,825	34.02	250.4									
Mack (SAMN16231354)	1/2a	12		I	SRR12695184	3,355,556	136.36		C	CP062126	2,864,720	38.05	154.9	2,851	2,762	2,738	89		117146–127872	10.7	Q	A118 (NC_003216; 5)
				N	SRR12695180	204,712	3,603.14												2175965–2197614	21.6	I	LP-064 (NC_041862; 3) LP-125 (NC_021781; 3)

a ST, sequence type; determined using MLST 2.0 (51).

b I, Illumina; N, Nanopore.

c Illumina and Nanopore read qualities were assessed using FastQC v0.11.7 (52).

d C, chromosome; P, plasmid.

e CDS, coding DNA sequences.

f Assembly statistics were generated using Quast v4.6.3 (53), BBMap v38.08 (54), and SAMtools v1.8 (55).

g Sum of rRNA, tRNA, and noncoding RNA (ncRNA) genes. All three contained 18 rRNA, 67 tRNA, and 4 ncRNA genes.

h Assemblies were queried for prophage regions using PHASTER (56).

i Q, questionable; I, incomplete.

### Data availability.

The sequencing data and assemblies for these bacterial strains are located under BioProject PRJNA664209 (the BioSample, SRA, and GenBank accession numbers are in Table 1).
